# The Influence of Heat Treatment Parameters on the Microstructure and Hardness of High-Chromium Alloy Steel X46Cr13

**DOI:** 10.3390/ma18174183

**Published:** 2025-09-05

**Authors:** Natalia Przyszlak, Tomasz Wróbel

**Affiliations:** Department of Foundry Engineering, Silesian University of Technology, 7 Towarowa Street, 44-100 Gliwice, Poland; tomasz.wrobel@polsl.pl

**Keywords:** high chromium steel, heat treatment, microstructure, hardness

## Abstract

This study presents the results of research on the influence of heat treatment parameters—namely, temperature, holding time, and quenching medium (air, water, oil)—on the microstructure and hardness of high-chromium alloy steel X46Cr13. The research included hardening heat treatment of X46Cr13 steel, hardness measurements, and microstructural analysis using light microscopy and scanning electron microscopy (SEM), supported by EDS chemical composition analysis. Based on the conducted studies, it was found that increasing the austenitizing temperature and time results in higher hardness of the X46Cr13 high-chromium alloy steel, regardless of the quenching medium used. Due to the negligible influence of the quenching medium on the hardness of the steel under the analyzed heat treatment conditions, air hardening is recommended, as it reduces quenching-induced stresses compared to water quenching.

## 1. Introduction

Among many alloy steels, corrosion-resistant steels are among the most important. Corrosion is a phenomenon involving the progressive degradation of metallic materials due to chemical or electrochemical interaction with a chemically active environment. As a result of corrosion processes, there is a continuous reduction in the effective cross-section of machine components during operation, accompanied by an increase in stress without any change in load. Corrosion can occur through various mechanisms and may cause uniform or localized damage [1,2,3,4,5].

In addition to chemical (anti-corrosion) properties, corrosion-resistant steels are also required to possess certain mechanical, physical, and functional properties. One of the key elements that actively enhances the corrosion resistance of steel is chromium. High-chromium steels are resistant to corrosion in various environments, such as atmospheric air, natural water, steam, aqueous alkaline solutions, acids, and salts—excluding iodides and chlorides [3,6,7,8].

Due to the critical influence of chromium on corrosion resistance, this element is also the main alloying addition in the group of high-chromium martensitic steels [3,9,10,11].

Martensitic corrosion-resistant steels have a chemical composition similar to ferritic steels but are characterized by a higher carbon content, ranging from 0.08 to 1.2 wt.% C. At temperatures above 1050 °C, the microstructure of martensitic steels is austenitic or austenitic with carbides, and during cooling the austenite transforms into martensite. An increased carbon content in Fe–Cr alloys promotes the expansion of the stability range of the γ-phase in the phase equilibrium diagram toward higher Cr concentrations. As a result, high-chromium steels attain a γ-phase solid solution structure at elevated temperatures. A chromium content above 10 wt.% provides such high hardenability that even air cooling ensures the formation of a martensitic structure, and these steels are considered self-hardening. After quenching, the steel is tempered to increase its ductility and impact toughness. The tempering process of these steels can be modified by adding V, Nb, Si, or W, while improvement in corrosion properties is achieved by adding Mo and Ni. These elements, on the other hand, by lowering the Ms temperature, may prevent the full formation of a martensitic structure; therefore, they should be added in a limited amount. To compensate for their undesirable effect in this regard, austenite-forming elements such as Ni, Co, Mn, or Cu should be added simultaneously. Carbon is an element that significantly affects the structure of martensitic steels. Chromium in Fe–Cr alloys has a higher affinity for carbon than iron, and therefore MxCy carbides are formed, the type of which depends on the Cr/C ratio. At a low Cr/C ratio, alloyed cementite (Fe, Cr)_3_C is formed. As the chromium concentration increases, (Cr, Fe)_7_C_3_ carbides with a complex hexagonal lattice are precipitated. At a high Cr/C ratio, the (Cr, Fe)_23_C_6_ carbide with a complex cubic lattice becomes stable [12,13,14,15,16].

The dynamic development of many industrial sectors creates a constant need for actions focused primarily on three aspects: process simplification, time efficiency, and cost reduction. These objectives also apply to the machine-building industry, where there is a growing demand for products with special properties—such as high resistance to abrasive wear and corrosion—manufactured from expensive and hard-to-access materials [17].

Often, however, the high requirements regarding performance properties apply only to those surfaces of castings that are directly exposed to destructive external environments (working surfaces), and therefore are subject to wear. Alloy steels, including corrosion-resistant steels, are materials with excellent mechanical and corrosion-resistant properties. Numerous scientific publications indicate that layered castings have been effectively produced, where such steels formed the working part, while materials like flake graphite grey cast iron were used for the load-base section. According to the definition, layered castings consist of at least two materials, of which at least one is produced using a casting technique, and the surface of the connection between them is as large as possible. If the number of components is limited to two, such castings are called bimetals. Layered castings consist of two main parts (layers): the working part and the base part. The base layer usually serves as the structural support of the casting and is made from a cheaper material, most often a common casting alloy that does not require high functional properties, e.g., unalloyed steel, gray cast iron, or aluminum and its alloys. Different criteria apply to the material of the working layer, which is the part of the casting exposed to harmful external factors and thus subject to wear. Materials with enhanced properties—such as corrosion or mechanical resistance—are selected for the working layer. An example of such a material is X46Cr13 steel [18,19,20,21,22,23].

Referring to the phenomenon of self-hardening in steel, it was assumed that it might be possible to combine bimetal casting technology with a heat treatment process carried out directly within the casting mold. Previous studies of X46Cr13 steel in the area of heat treatment have shown that the fraction and composition of chromium carbides are determined by the temperature and duration of heat treatment. It has been demonstrated that the highest hardness and corrosion resistance of the tested steel are obtained after water quenching. This results from the uniform distribution of chromium and carbon in the solid solution. Air cooling of the steel, on the other hand, may lead to a reduction in corrosion resistance [24].

Accordingly, as part of preliminary research aimed at evaluating the effectiveness of hardening the selected grade of X46Cr13 steel integrated with bimetal casting technology, reference hardness values were determined for this steel grade, achievable through conventional heat treatment methods.

## 2. Materials and Methods

The main objective of the study was to evaluate the effect of varying heat treatment parameters on the microstructure and hardness of martensitic stainless steel X46Cr13 [25] A conventional heat treatment was carried out on X46Cr13 steel using 5 mm thick sheets in the annealed condition. Soft annealing of the sheets, involving slow furnace cooling from a temperature of 850 °C, is a typical heat treatment process applied by X46Cr13 steel manufacturers and is commonly referred to as the delivery condition.

According to the [26], after annealing, X46Cr13 steel should meet the following mechanical property criteria: UTS ≤ 780 MPa, YS 0.2 ≤ 345 MPa, EL ≥ 12%, hardness ≤ 250 HV (22 HRC).

For the purpose of designing the aforementioned heat treatment, in addition to literature data [7], the results of the chemical composition analysis of the tested steel—performed using a LECO GDS 500A glow discharge optical emission spectrometer (LECO Corporation, St. Joseph, MI, USA)—were used, along with phase composition predictions obtained from the Thermo-Calc TCFE 11 and JMatPro v.12 software. Based on this information, the following ranges for the variable factors in the steel hardening process were adopted:

- Austenitizing temperature T_γ_ = 950–1150 °C, in 50 °C increments;
- Holding time in T_γ_:t_γ_ = 150–750 s, in 150 s increments;
- Cooling rate from T_γ_:V_cooling_ depended on the use of different cooling media, i.e., still air (A), oil (O), and water (W).

Experimental plan for hardening of X46Cr13 steel is presented in Table 1, whereas chemical composition of X46Cr13 is presented in Table 2.

Microstructural and phase composition analyses were conducted using scanning electron microscopy (SEM) INSPECT F (FEI Technologies Inc., Hillsboro, OR, USA) with an energy X-ray dispersive spectrometer (EDS).

Hardness measurements were carried out in five selected areas on the surface of the steel sheet in both the delivery condition and after hardening. A universal hardness tester SBRV–1000 Sunpoc (Guizhou Sunpoc Tech Industry Co., Ltd., Guiyang, China) was used. Measurements were performed using the Brinell method [27] with a 5 mm cemented carbide ball and a 7500 N load, as well as the Rockwell method [28] with a 120° diamond cone and a 1471 N load. In both cases, the results were converted to the Vickers scale.

The test results were statistically processed using multiple regression analysis in Statistica v. 13.3 software, and the significance of the derived empirical relationships was verified using the Fisher test. In this way, the following relationship (1) was determined:

HV = f(T_γ_, t_γ_) (1)

for each V_cooling_.

## 3. Results and Discussion

**Figure 1:**
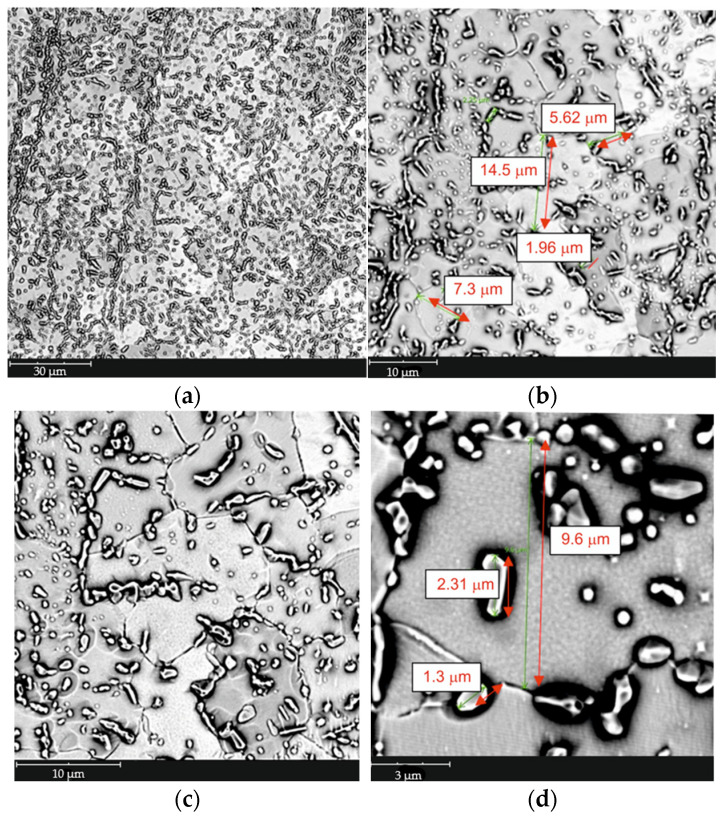
Microstructure of X46Cr13 steel in the annealed condition—Cr(Fe) carbide in a ferritic matrix, SEM, magnification: (**a**) 2000×; (**b**) 5000×; (**c**) 8000×; (**d**) 20,000×.

**Figure 2:**
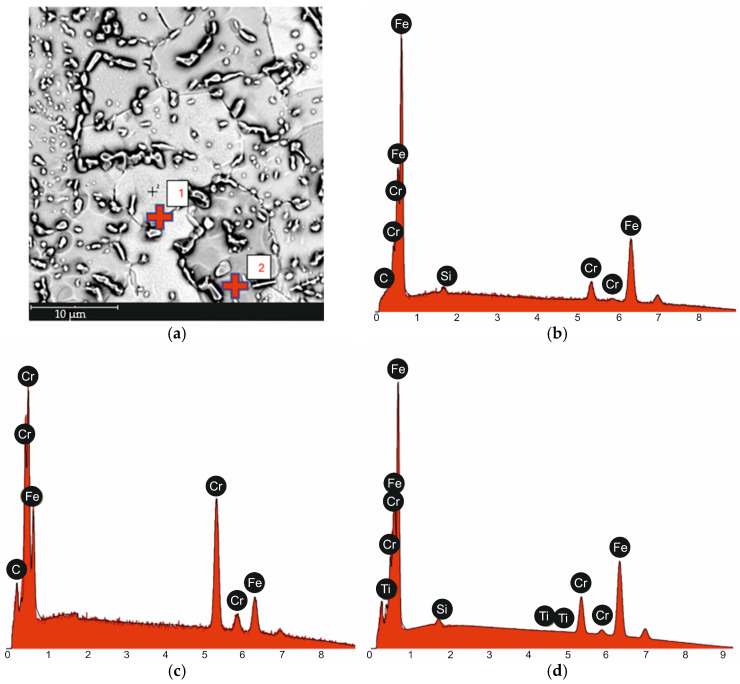
Microstructure of X46Cr13 steel in the annealed condition with marked points of EDS spot analysis (**a**), EDS analysis result at point 1 (**b**), EDS analysis result at point 2 (**c**), EDS surface analysis result (**d**).

**Figure 3:**
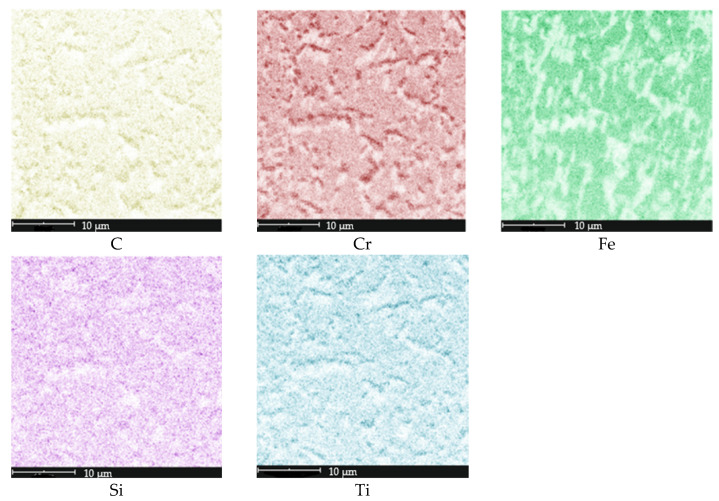
Surface elemental distribution in the microstructure from Figure 2a.

**Figure 4:**
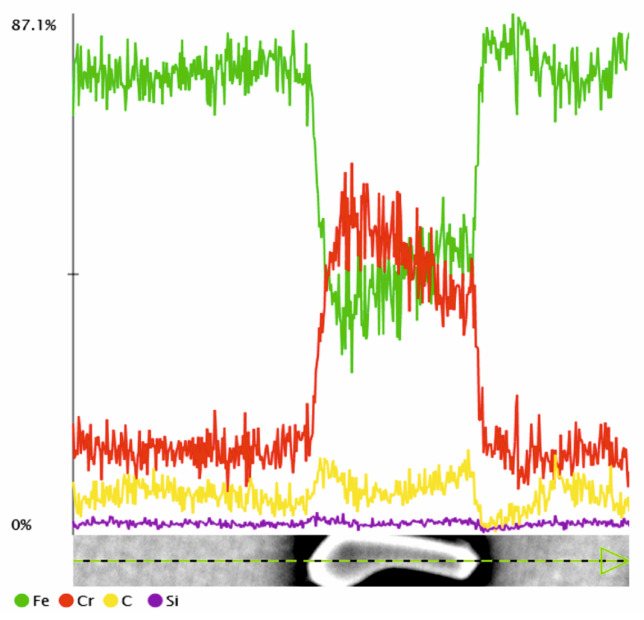
Line distribution of elements in the Cr(Fe) carbide within the ferritic matrix.

Based on the conducted metallographic studies, it was found that in the delivery condition, X46Cr13 steel is characterized by a microstructure consisting of very fine Cr(Fe) carbides with an average size of approximately 2 µm, embedded in a ferritic matrix with an average grain size of about 10 µm (Figure 1, Figure 2, Figure 3 and Figure 4 and Table 3). The Cr(Fe) carbides are distributed both within the ferrite grains and along their boundaries.

**Figure 5:**
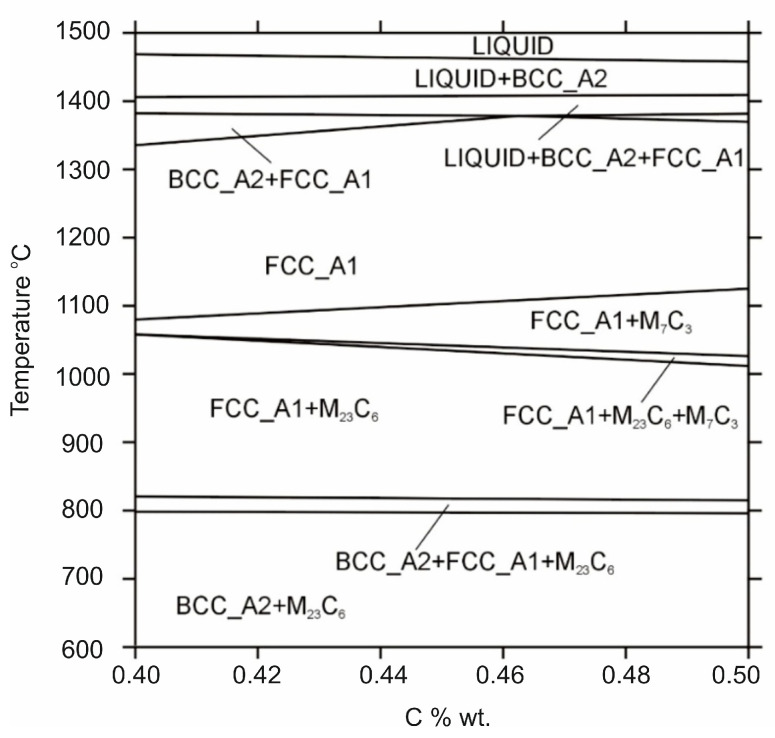
Phase equilibrium diagram of the investigated X46Cr13 steel determined using the Ther-mo-Calc program: LIQUID—liquid phase, FCC_A1—austenite, BCC_A2—ferrite, M_7_C_3_ and M_23_C_6_—Cr(Fe) carbides [30].

**Figure 6:**
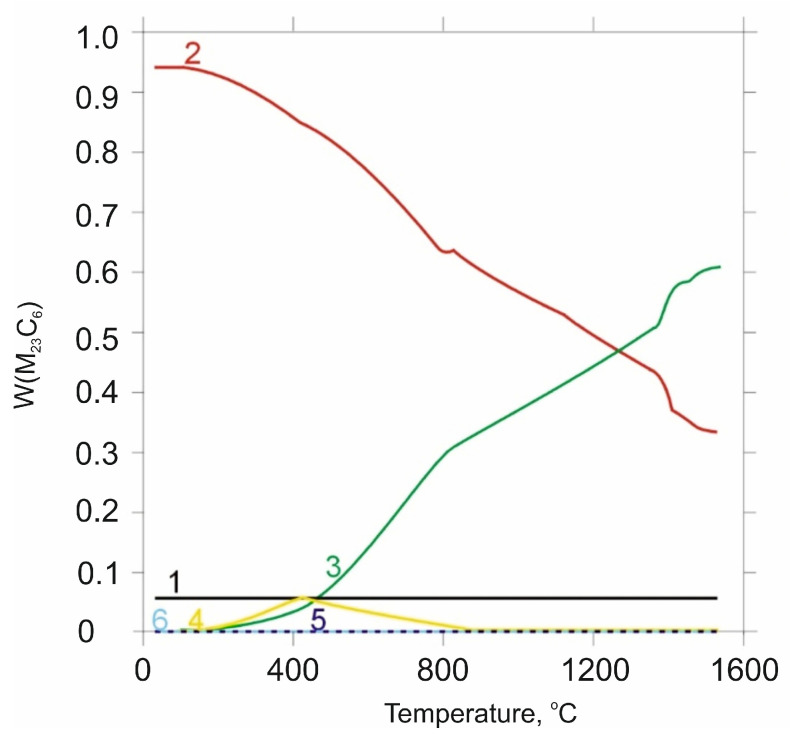
Mass fraction of elements in the M23C6 phase predicted using the Thermo-Calc program: 1—C; 2—Cr; 3—Fe; 4—Mn; 5—Ni; 6—Si. Conducted based on the chemical composition according to Table 2.

Based on the analysis of literature data [8,29] and the microstructural phase prediction performed using the Thermo-Calc TCFE 11 software, it was determined that in the annealed state, the carbide phase in X46Cr13 steel consists of the (Cr,Fe)_23_C_6_ phase (Figure 5 and Figure 6).

The phase composition of the microstructure in the delivery condition allows the material to achieve a hardness of 220 HV, which is within the limits specified by the aforementioned standard.

The development of hardness through the selection of cooling rate in the hardening process is primarily determined by the type of matrix formed for the Cr(Fe) carbides. Analysis of the CCT diagram according to [29] indicates that, depending on the cooling rate after austenitizing, the resulting microstructure will consist of Cr(Fe) carbides embedded in various matrices—ferritic, pearlitic, martensitic, or a mixture of these. Furthermore, based on the CCT diagram, it can be concluded that in order to obtain a matrix in which martensite is the dominant phase surrounding the Cr(Fe) carbides, X46Cr13 steel must be cooled in the temperature range of 800–500 °C at a rate of V_8_/_5_ ≥ 0.5 °C/s.

**Figure 7:**
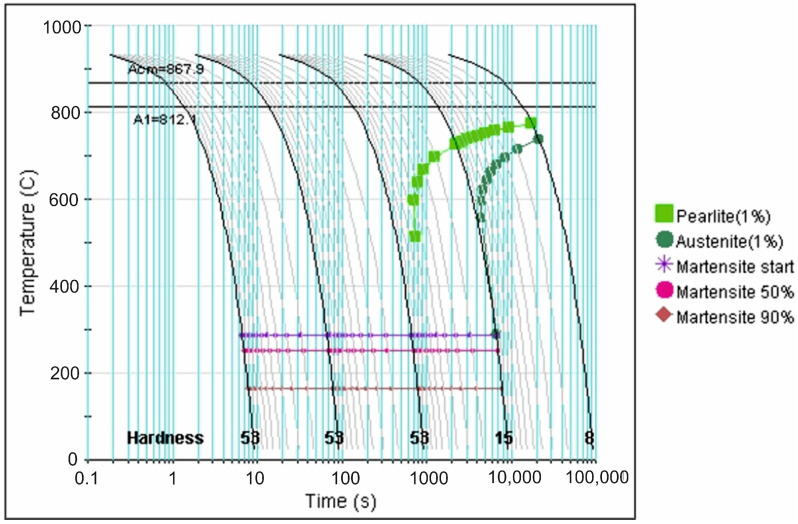
TTTc diagram (according to the JMatPro v.12 program) for X46Cr13 steel with the chemical composition from Table 2 and an austenitizing temperature of 950 °C.

**Figure 8:**
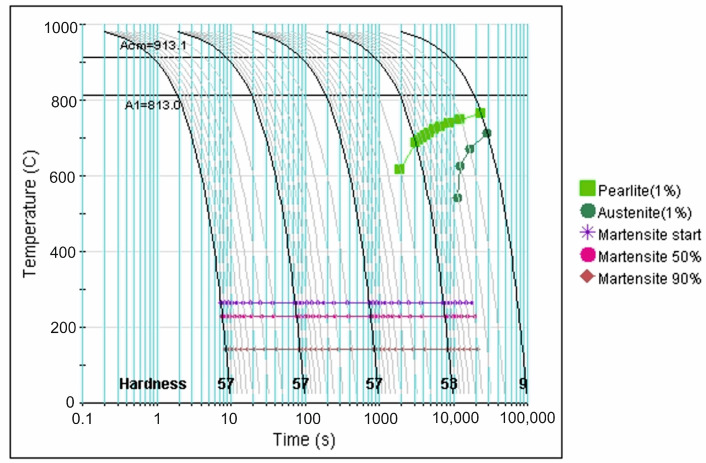
TTTc diagram (according to the JMatPro v.12 program) for X46Cr13 steel with the chemical composition from Table 2 and an austenitizing temperature of 1000 °C.

**Figure 9:**
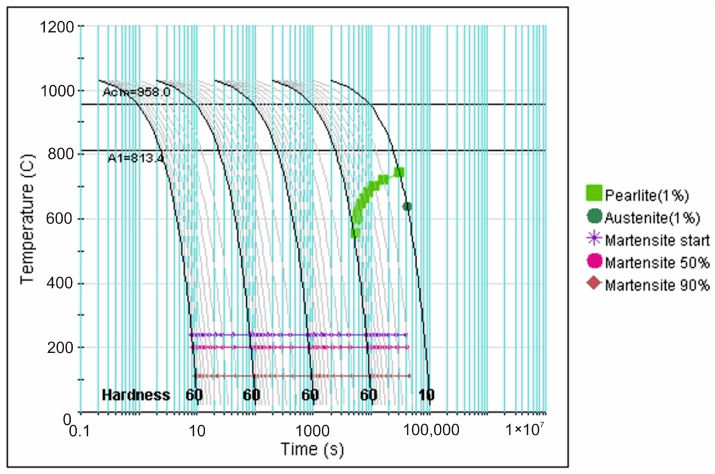
TTTc diagram (according to the JMatPro v.12 program) for X46Cr13 steel with the chemical composition from Table 2 and an austenitizing temperature of 1050 °C.

**Figure 10:**
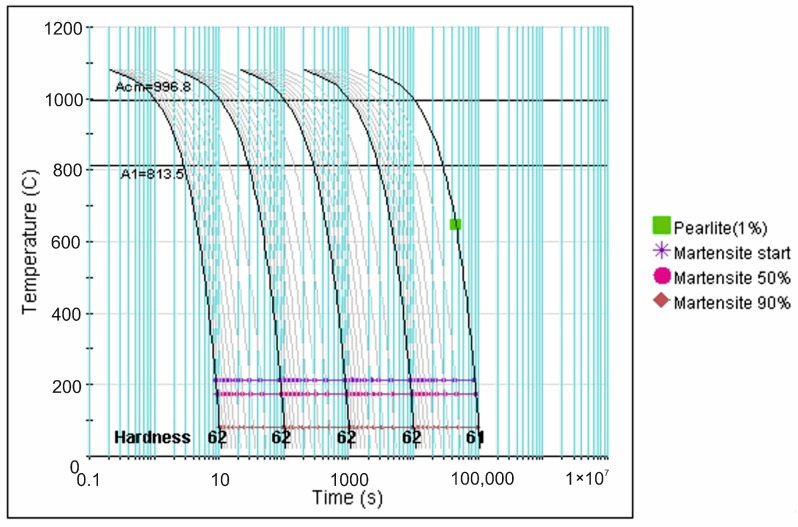
TTTc diagram (according to the JMatPro v.12 program) for X46Cr13 steel with the chemical composition from Table 2 and an austenitizing temperature of 1100 °C.

**Figure 11:**
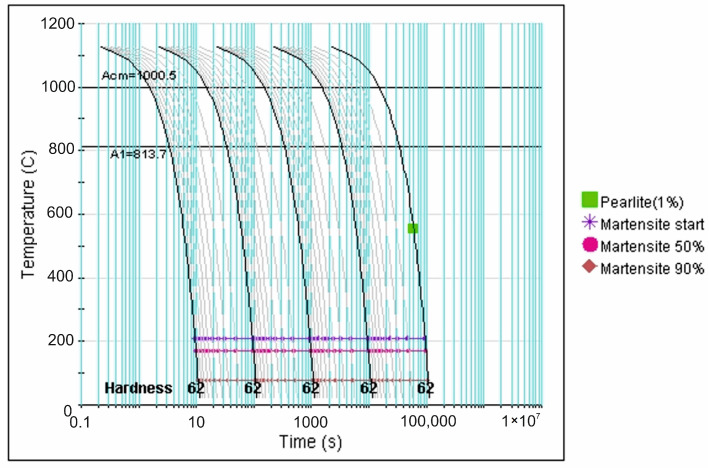
TTTc diagram (according to the JMatPro v.12 program) for X46Cr13 steel with the chemical composition from Table 2 and an austenitizing temperature of 1150 °C.

More precise data in this regard is provided by analysis of the CCT diagrams generated using the JMatPro v.12 software (Figure 7, Figure 8, Figure 9, Figure 10 and Figure 11) for the investigated steel with a precisely determined chemical composition, as presented in Table 2. It was found that increasing the austenitizing temperature allows for a higher maximum hardness to be achieved in the hardening process of X46Cr13 steel—up to 62 HRC (748 HV). Moreover, obtaining martensite as the clearly dominant matrix phase for Cr(Fe) carbides in the microstructure of the examined X46Cr13 steel requires cooling in the 800–500 °C range at a minimum rate ranging from approximately 0.6 to 0.01 °C/s. The higher cooling rate is necessary at the lower austenitizing temperature of 950 °C, while the lower rate suffices at the higher austenitizing temperature of 1150 °C.

According to the literature data [8], the austenitizing temperature for X46Cr13 steel should fall within the range of 950 to 1050 °C. However, due to the results obtained using the JMatPro v.12 software—which predicted a positive influence of increased austenitizing temperature on the final hardness of the steel, as well as a rightward shift of the phase transformation curves (indicating a reduction in critical cooling rate)—the study also included T_γ_ = 1100 °C and 1150 °C.

Regarding the austenitizing time, standard [31] recommends 90 s of soaking per 1 mm of sheet thickness, which results in 450 s for a 5 mm thick sheet. To verify this assumption, the experimental plan included austenitizing times (t_γ_) in the range of ±300 s from the optimal time, in 150 s intervals.

In selecting the quenching media, the commonly used options in steel hardening were chosen—namely, oil (O) and water (W). Additionally, considering the high hardenability of X46Cr13 steel [2], and the fact that even relatively slow cooling rates may induce a martensitic transformation, still air (A) was selected as the third quenching medium.

**Figure 12:**
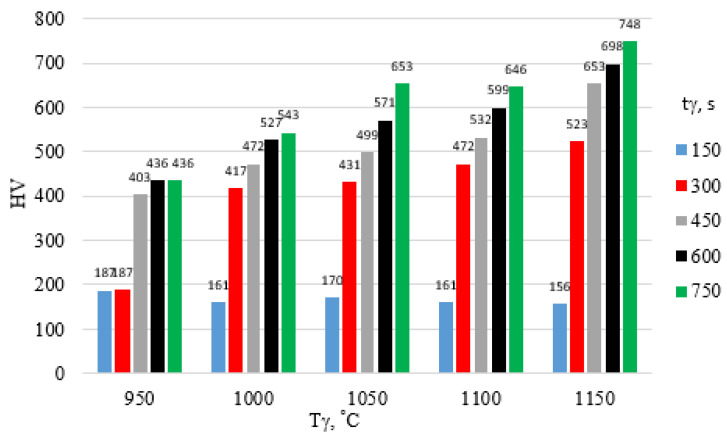
Influence of T_γ_ and t_γ_ on the HV hardness of X46Cr13 steel hardened in still air.

**Figure 13:**
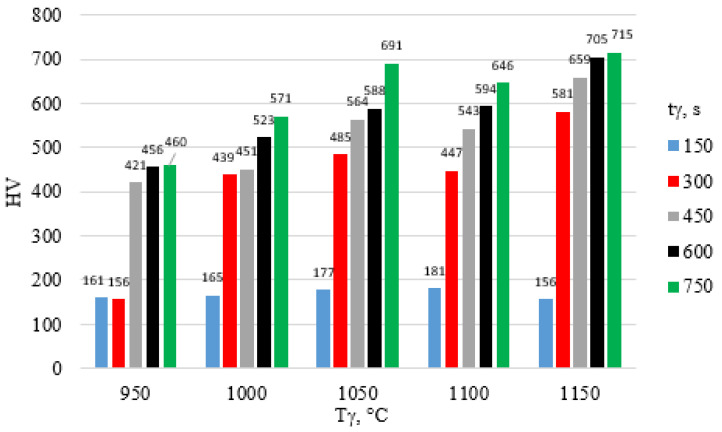
Influence of T_γ_ and t_γ_ on the HV hardness of X46Cr13 steel hardened in oil.

**Figure 14:**
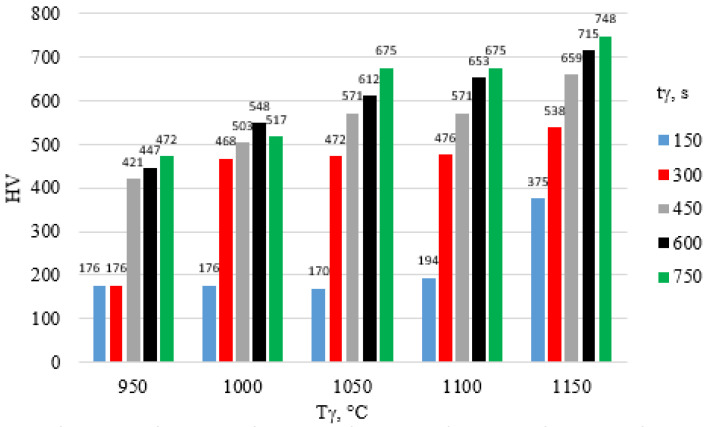
Influence of T_γ_ and t_γ_ on the HV hardness of X46Cr13 steel hardened in water.

The surface hardness of X46Cr13 steel after hardening is presented in Figure 12, Figure 13 and Figure 14. The highest hardness of the 5 mm thick steel sheet—62 HRC (approx. 750 HV)—was achieved during hardening in air and in water using an austenitizing temperature and time of 1150 °C and 750 s, respectively.

Table 4 presents the relationships HV = f(T_γ_, t_γ_) for each quenching medium, along with the corresponding statistical parameters, including the correlation coefficient R, adjusted R^2^, Fisher’s F-test value, and standard deviation s.

**Figure 15:**
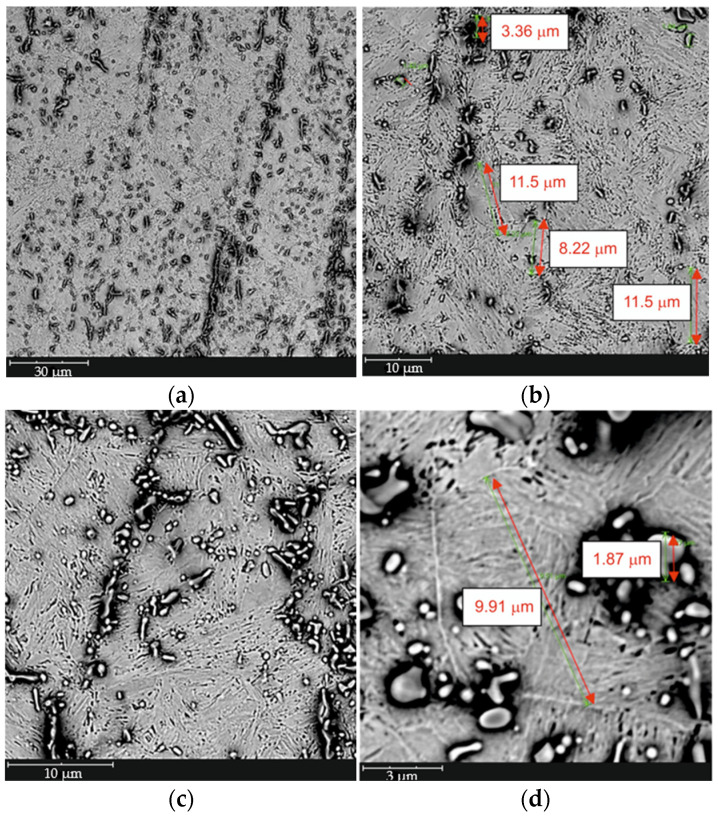
Microstructure of X46Cr13 steel after hardening from 1150 °C in air—Cr(Fe) carbide in a martensitic matrix, SEM, magnification: (**a**) 2000×, (**b**) 5000×, (**c**) 8000×, and (**d**) 20,000×.

**Figure 16:**
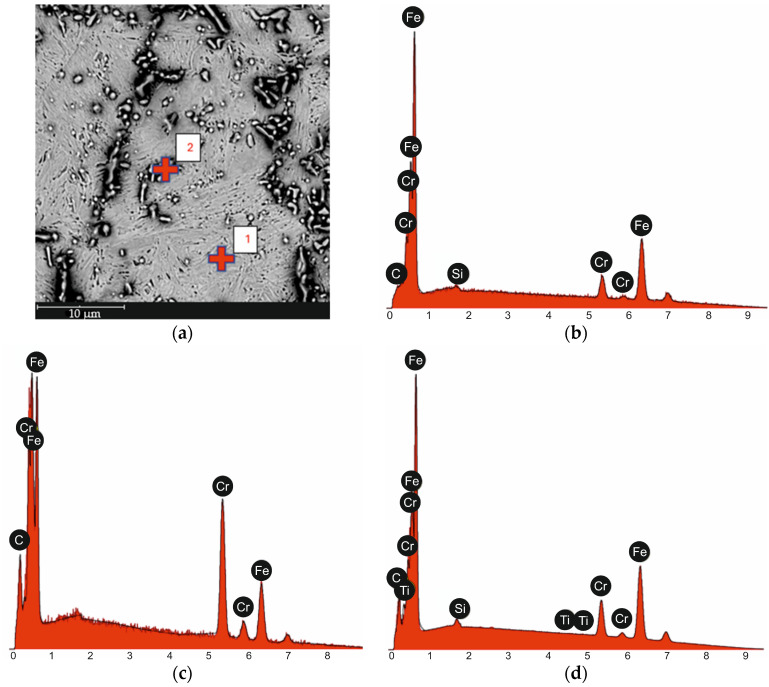
Microstructure of X46Cr13 steel in the hardened state with marked locations of point EDS analysis (**a**), EDS analysis result at point 1 (**b**), EDS analysis result at point 2 (**c**), surface EDS analysis result (**d**).

**Figure 17:**
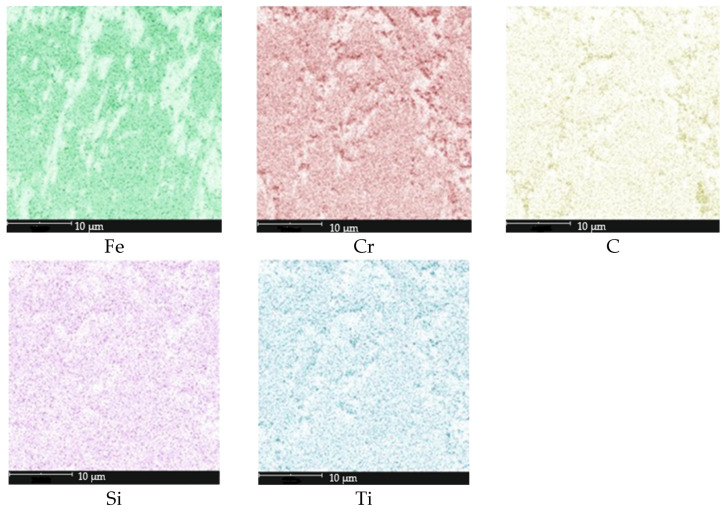
Surface elemental distribution in the microstructure from Figure 16a.

**Figure 18:**
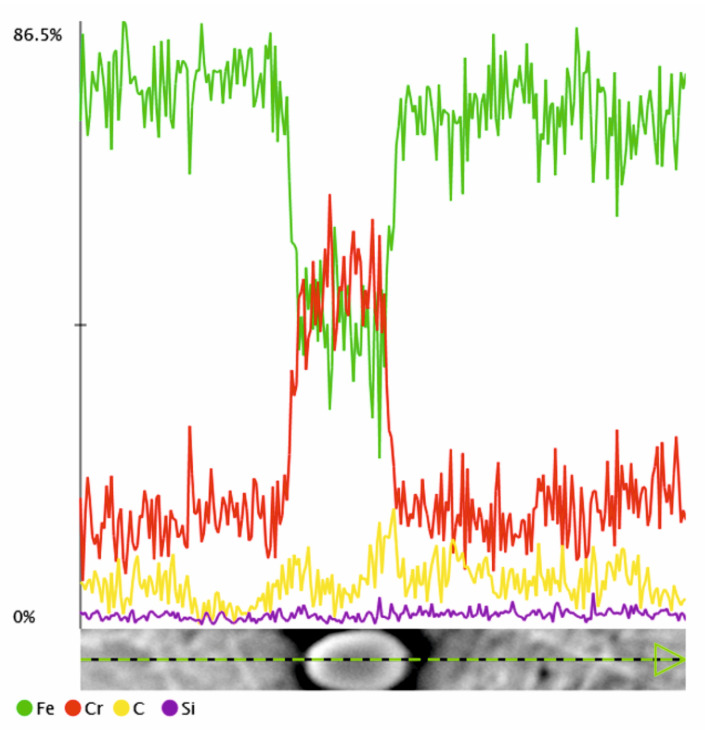
Line distribution of elements in the Cr(Fe) carbide within the martensitic matrix.

Figure 15 shows a representative microstructure of X46Cr13 steel after air hardening from 1150 °C with an austenitizing time of 750 s. It was found that this type of microstructure—composed of very fine Cr(Fe) carbides with an average size of approximately 2 μm, embedded in a fine-lath martensitic matrix located within prior austenite grains averaging around 10 μm in size (Figure 16, Figure 17 and Figure 18 and Table 5)—ensures that the hardness of X46Cr13 steel reaches approximately 750 HV. The type of carbide was also determined based on the analysis of the literature concerning the heat treatment of X46Cr13 steel [24,32,33,34]. Therefore, this microstructure should be considered appropriate in terms of hardening efficiency for the given steel grade. The (Cr, Fe)_23_C_6_ carbides are primarily distributed within the prior austenite grains, and more rarely along their boundaries.

**Figure 19:**
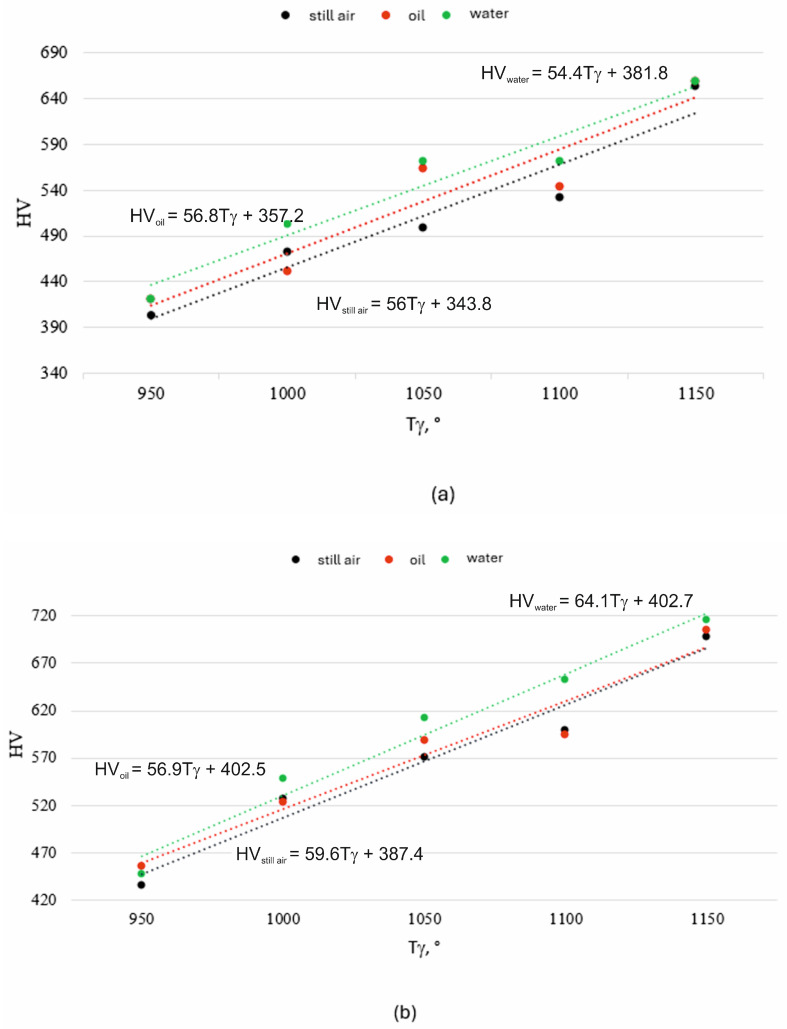

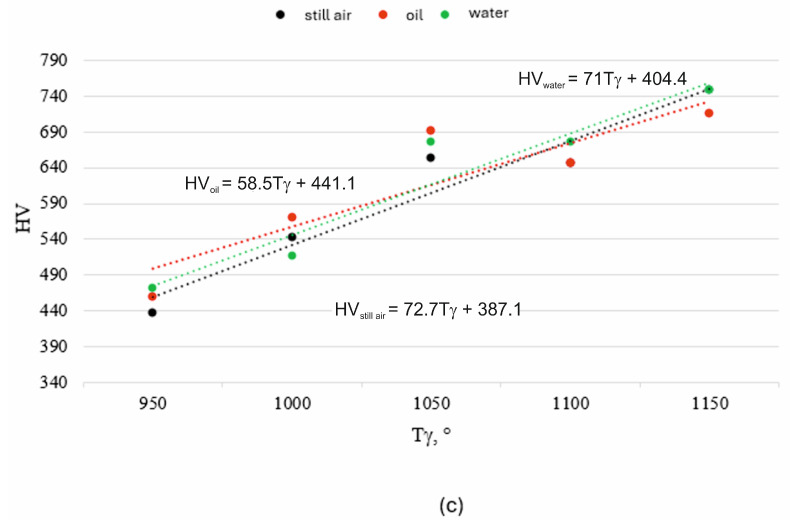
Relationship HV = f(T_γ_) at (**a**) t_γ_ = 450 s; (**b**) t_γ_ = 600 s; (**c**) t_γ_ = 750 s.

Summarizing the results at this stage of the preliminary research, it was found that the hardness of the high-chromium alloy steel X46Cr13 increases with rising austenitizing temperature and time, regardless of the quenching medium used in the hardening process. This is confirmed by the analysis of the slope angles (β) of the linear functions HV = f(T_γ_), determined based on the performed measurements for selected austenitizing times t_γ_ = 450, 600, and 750 s (Figure 19 and Table 6).

## 4. Conclusions

Based on the conducted studies, the following conclusions have been formulated:

- An increase in austenitizing temperature and time leads to higher hardness of the high-chromium alloy steel X46Cr13, regardless of the quenching medium used in the hardening process.
- The highest hardness of the 5 mm-thick high-chromium alloy steel X46Cr13 sheet—approximately 750 HV (62 HRC)—was achieved through air and water hardening at an austenitizing temperature and time of 1150 °C and 750 s, respectively. Therefore, for further considerations regarding layered casting technology, the value of 62 HRC will be adopted as the maximum achievable hardness.
- Proper selection of hardening parameters for the high-chromium alloy steel X46Cr13 allows the formation of a favorable microstructure composed of Cr and Fe carbides in a martensitic matrix, which is desirable in terms of achieving high hardness.
- Due to the negligible influence of the quenching medium on the hardness of high-chromium alloy steel X46Cr13 in the analyzed heat treatment, air hardening is recommended, as it reduces quenching-induced stresses compared to water hardening.
- The developed heat treatment variant can be applied in the production of cutting tools or casting molds made of X46Cr13 steel.

## Figures and Tables

**Table 1:** Heat treatment experimental plan for hardening of X46Cr13 steel.

t_γ_, s	150			300			450			600			750		
Cooling Medium	A	W	O	A	W	O	A	W	O	A	W	O	A	W	O
T_γ_, °C	950														
No.	1	2	3	4	5	6	7	8	9	10	11	12	13	14	15
T_γ_, °C	1000														
No.	16	17	18	19	20	21	22	23	24	25	26	27	28	29	30
T_γ_, °C	1050														
No.	31	32	33	34	35	36	37	38	39	40	41	42	43	44	45
T_γ_, °C	1100														
No.	46	47	48	49	50	51	52	53	54	55	56	57	58	59	60
T_γ_, °C	1150														
No.	61	62	63	64	65	66	67	68	69	70	71	72	73	74	75

**Table 2:** Chemical composition of X46Cr13 steel.

Element Concentrations, wt.%											
C	Cr	Ni	Mn	Mo	Co	Si	Al	Cu	V	W	P
0.43	13.6	0.125	0.375	0.015	0.011	0.383	0.003	0.069	0.099	0.021	0.025

**Table 3:** Results of EDS analysis for point 1 and 2 from Figure 2a.

Measurement Point	Element	%at.	%wt.
1 from Figure 2a	Fe	89.0	90.0
	Cr	10.3	9.7
	Si	0.7	0.4
	C	0.0	0.0
2 from Figure 2a	Cr	52.1	57.2
	Fe	33.1	39.0
	C	14.9	3.8
EDS Surface Analysis Result from Figure 2a	Fe	71.1	81.1
	Cr	14.2	15.1
	C	14.0	3.4
	Si	0.7	0.4
	Ti	0.0	0.0

**Table 4:** Summary of statistical analysis results performed using multiple regression for the three quenching media in the hardening process of X46Cr13 steel.

	Still Air	Oil	Water
HV = f(T, t)	HV = 1.02 T + 0.71 t − 943.2	HV = 1.1 T + 0.71 t − 1009.74	HV = 1.22 T + 0.69 t – 1087.96
R	0.91	0.89	0.92
R2	0.83	0.79	0.84
s	79.64	91.25	75.93
F	54.79	42.03	56.64
F_0.5_	3.12		

**Table 5:** Results of EDS analysis for point 1 and 2 from Figure 16a.

Measurement Point	Element	%at.	%wt.
1 from Figure 16a	Fe	86.0	87.5
	Cr	12.9	12.2
	C	0.8	0.2
	Si	0.3	0.2
2 from Figure 16a	Cr	39.6	45.7
	Fe	39.2	48.6
	C	21.3	5.7
EDS Surface Analysis Result from Figure 16a	Fe	67.8	80.0
	Cr	13.8	15.2
	C	17.8	4.5
	Si	0.5	0.3
	Ti	0.1	0.1

**Table 6:** Values of the slope angles β relative to the *x*-axis for the HV = f(T_γ_) linear relationships at a given austenitizing time t.

Austenitizing Time t_γ_, s	Cooling Medium	β, °
450	Still air	48.24
	Oil	48.64
	Water	47.41
600	Still air	50.00
	Oil	48.69
	Water	52.04
750	Still air	55.48
	Oil	49.48
	Water	54.85

## Data Availability

The original contributions presented in this study are included in the article. Further inquiries can be directed to the corresponding author.
